# Rapid synaptic potentiation within the anterior cingulate cortex mediates trace fear learning

**DOI:** 10.1186/1756-6606-5-6

**Published:** 2012-02-03

**Authors:** Giannina Descalzi, Xiang-Yao Li, Tao Chen, Valentina Mercaldo, Kohei Koga, Min Zhuo

**Affiliations:** 1 Department of Physiology, Faculty of Medicine, University of Toronto, 1 King's College Circle, Toronto, Ontario M5S 1A8, Canada; 2 Center for Neuron and Disease, Frontier Institute of Science and Technology, Xi'an Jiaotong University, 28 Xianning West Road, Xian, Shaanxi 710049, China

**Keywords:** fear learning, memory consolidation, ACC, GluA1, NMDA, Ca^2+ ^permeable AMPARs

## Abstract

Although the cortex has been extensively studied in long-term memory storage, less emphasis has been placed on immediate cortical contributions to fear memory formation. AMPA receptor plasticity is strongly implicated in learning and memory, and studies have identified calcium permeable AMPA receptors (CP-AMPARs) as mediators of synaptic strengthening. Trace fear learning engages the anterior cingulate cortex (ACC), but whether plastic events occur within the ACC in response to trace fear learning, and whether GluN2B subunits are required remains unknown. Here we show that the ACC is necessary for trace fear learning, and shows a rapid 20% upregulation of membrane AMPA receptor GluA1 subunits that is evident immediately after conditioning. Inhibition of NMDA receptor GluN2B subunits during training prevented the upregulation, and disrupted trace fear memory retrieval 48 h later. Furthermore, intra-ACC injections of the CP-AMPAR channel antagonist, 1-naphthylacetyl spermine (NASPM) immediately following trace fear conditioning blocked 24 h fear memory retrieval. Accordingly, whole cell patch clamp recordings from c-fos positive and c-fos negative neurons within the ACC in response to trace fear learning revealed an increased sensitivity to NASPM in recently activated neurons that was reversed by reconsolidation update extinction. Our results suggest that trace fear learning is mediated through rapid GluN2B dependent trafficking of CP-AMPARs, and present *in vivo *evidence that CP-AMPAR activity within the ACC immediately after conditioning is necessary for subsequent memory consolidation processes.

## Background

Long term potentiation (LTP) of central synapses is believed to be the basic mechanism that drives memory storage within the brain [1,2]. Although a critical role for the cerebral cortex in remote fear memory recall has been established [3], little is known regarding immediate cortical contributions to fear memory formation. Much effort instead has focused on the amygdala, where animal studies revealed that associative fear conditioning, which pairs an arbitrary conditioning stimulus (CS) with a noxious one (US), induces changes in excitatory glutamatergic transmission [4-6], and requires postsynaptic GluA2 expression for memory maintenance [7]. Evidence suggests however that in addition to the amygdala, cortical structures also mediate fear learning. In humans, trace fear conditioning, which introduces a time interval between the CS and the US, activates several brain areas including the amygdala, hippocampus, medial prefrontal cortex (mPFC), and the anterior cingulate cortex (ACC) [8,9]. The ACC is involved in the processing of pain, emotion, and threat related stimuli [10,11], and we recently found a trace fear memory enhancement in mice overexpressing Ca^2+ ^⁄ calmodulin-dependent protein kinase IV (CaMKIV), that corresponded with enhancements of ACC LTP in layer II/III pyramidal neurons [12]. In rats, trace fear conditioning induces ACC c-fos expression, and visual distraction during the time interval separating the CS and US prevents fear memory and c-fos expression [13].

Glutamatergic synapses in the ACC are plastic [14-16], and the *N*-methyl-D-aspartate (NMDA) receptors are critical for LTP induction within the ACC [17]. The GluN2B subunit in particular has been found to be a critical mediator of pain induced alterations within the ACC [18], and forebrain overexpression of GluN2B in mice enhances contextual and auditory fear memory [19]. We previously showed that LTP induction within the ACC corresponds with postsynaptic upregulation of AMPA receptor GluA1 subunits [15,20]. Interestingly, AMPA receptor plasticity is strongly implicated in learning and memory [5,21], and several studies suggest that calcium permeable AMPA receptors (CP-AMPARs) mediate synaptic strengthening [22-24]. In particular, in the CA1 region of the hippocampus, transient increases of CP-AMPARs were observed in response to LTP induction through theta burst stimulation [23] and pairing protocols [24]. However whether such rapid plastic events occur within the cortex in response to trace fear learning, and whether GluN2B subunits are required remains unknown. In the present study, we used integrative methods, including behavioral, pharmacological, biochemical, and electrophysiological, to determine if plasticity related events occur within the ACC during trace fear learning.

## Results

### Trace fear learning upregulates membrane AMPA receptor GluA1 subunits within the ACC

**Figure 1:**
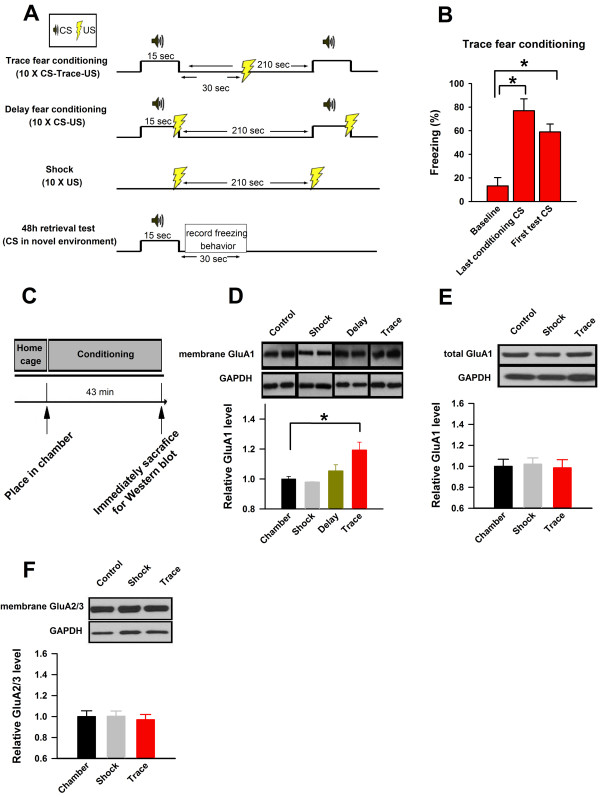
**Trace fear upregulates membrane AMPA receptor GluA1 subunits within the ACC**. **(A) **We exposed adult (8-12 wks) C57 mice to one of 4 conditions: trace fear conditioning, delay fear conditioning, shock only, or exposure to the chamber (all treatments lasted 43 min). **(B) **48 h later, in a novel environment, mice exposed to trace fear conditioning exhibit robust freezing behavior in response to the CS. **(C) **We performed western blot analysis of ACC samples extracted immediately following conditioning. **(D) **Mice exposed to trace fear conditioning show a significant upregulation of membrane bound GluA1 in the ACC (chamber: n = 8; trace fear: n = 8; shock: n = 6; delay: n = 8; *F *= 6.70). **(E) **Total GluA1 levels within the ACC were not affected by trace fear conditioning (chamber: n = 6; shock:, n = 4, trace fear: n = 6; *F *= 0.06.. **(F) **Membrane bound GluA2/3 expression levels were not affected by trace fear conditioning (chamber: n = 5; shock:, n = 4, trace fear: n = 5; *F *= 0.113. (* *P < 0.02*).

In order to investigate trace fear learning induced alterations within the ACC, we analyzed the ACC of mice following exposure to a trace fear conditioning paradigm that pairs an auditory conditioning tone (CS) with a foot shock (US), with a 30 sec interval (trace) separating the CS from the US (Figure 1A). This paradigm reliably induces freezing behaviour in mice exposed to the CS in a novel context 48 h later (Figure 1B). To determine if membrane bound AMPA receptor expression is altered in the ACC in response to trace fear conditioning, we extracted the ACC of mice immediately after conditioning, and performed Western blot analysis (Figure 1C). We compared the expression levels of membrane AMPA receptor GluA1 subunits in the ACC of adult (8-12 wks) C57 mice exposed to one of four conditions: trace fear conditioning (10 × CS-trace-US), shock only (10 × US), delay fear conditioning (10 × CS-US), or exposure to the conditioning chamber. Remarkably, trace fear conditioning induced a rapid, significant upregulation of membrane bound GluA1subunits in the ACC (chamber: 1 ± 0.02; trace fear: 1.19 ± 0.05 times the chamber alone value; shock: 1.00 ± 0.07 times the chamber alone value; delay: 1.05 ± 0.07 times the chamber alone value; one-way ANOVA, *P *= 0.002; Figure 1D). Importantly, total GluA1 levels within the ACC remained unchanged in mice exposed to trace fear conditioning (Figure 1E), and we observed similar levels of membrane bound GluA2/3 (Figure 1F), suggesting a membrane upregulation that was limited to the GluA1 subunit of AMPA receptors. These results suggest that trace fear learning may be mediated through an upregulation of synaptic ACC AMPA receptors containing the GluA1 subunit.

### Activation of NMDA receptor GluN2B subunits is required for the induction of trace fear memory

**Figure 2:**
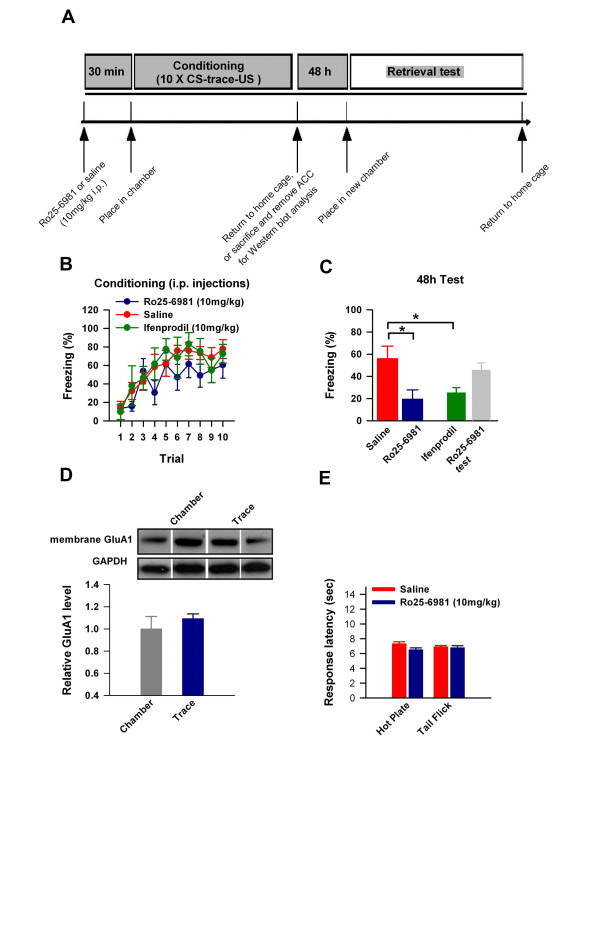
**NMDA receptor GluN2B subunit activity is required for trace fear memory**. **(A) **We exposed mice to i.p. injections of either Ro25-6981 or ifenprodil (10 mg/kg @ 2 mg/ml), or saline, 30 min prior to trace fear conditioning and assessed freezing behavior in response to the CS in a new context 48 h later. **(B-C) **Mice exposed to either Ro25-6981 or ifenprodil prior to training exhibited normal learning behavior but a robust reduction in freezing behavior in response to the CS compared to mice exposed to saline, or to mice exposed to Ro25-6981 30 min prior to testing. **(D) **Treatment with Ro25-6981 30 min prior to training completely blocked trace fear induced membrane GluA1 upregulation in the ACC (saline: n = 7; Ro25-6981: n = 8; ifenprodil: n = 6; Ro25-6981 at test: n = 5; *F *= 6.64). **(E) **Mice displayed similar response latencies in the hot plate (55°C) and tail flick assays when treated with Ro25-6981 or saline 30 min before testing. (* *P < 0.02*).

To investigate if GluN2B subunit activity was necessary for trace fear memory, we used two selective NMDA receptor GluN2B antagonists: Ro25-6981 and Ifenprodil, which have been repeatedly used to assess GluN2B function [17,25]. We exposed mice to intraperitoneal (i.p.) injections of Ro-25-6981 (10 mg/kg), Ifenprodil (10 mg/kg), or saline, 30 min prior to trace fear conditioning and assessed freezing behaviour in response to the CS in a new context 48 h later (Figure 2A). A distinct trend can be observed in the latter half of training where mice exposed to GluN2B antagonists displayed attenuated freezing levels; however it did not reach statistical significance (Figure 2B). When tested in a new context 48 h later, mice exposed to saline displayed robust freezing in response to the CS (Figure 2C). In contrast, mice treated with either Ro25-6981 or Ifenprodil prior to training exhibited a marked reduction in freezing behaviour in response to the CS compared to mice exposed to saline, or to mice exposed to Ro25-6981 30 min prior to testing (saline: 56 ± 7%; Ro25-6981: 19 ± 9%; ifenprodil: 23 ± 4%; Ro25-6981 at test: 49 ± 10%; one-way ANOVA, *P *= 0.005; Figure 2C), indicating that GluN2B activity is necessary for trace fear learning.

### Trace fear learning induced membrane GluA1 upregulation is GluN2B dependent

Our results revealed that trace fear conditioning rapidly upregulates GluA1 subunits within the ACC, and that NMDA receptor GluN2B subunit activity is required for trace fear conditioning. We thus next sought to determine if treatment with Ro25-6981 prior to trace fear conditioning affects learning induced upregulation of membrane GluA1 within the ACC. We exposed mice to i.p. injections of Ro25-6981 (10 mg/kg i.p.) and either exposed them to trace fear conditioning, or to the chamber alone. Remarkably, treatment with Ro25-6981 prior to training completely blocked trace fear induced membrane GluA1 upregulation within the ACC (chamber: 1.0 ± 0.1; trace fear: 1.1 ± 0.1 times the chamber alone value; Figure 2D), indicating that the downstream target of learning induced GluN2B activity is the AMPA receptor GluA1 subunit. Importantly, the effects of Ro25-6981 treatment on trace fear learning are not due to any analgesic effects that may impair CS-US associations, as i.p. injections of Ro25-6981 had no effect on acute pain nociception as evidenced by similar nociceptive thresholds in hot plate and tail flick assays (Figure 2E).

### GluN2B subunits within the ACC are required for trace fear memory

**Figure 3:**
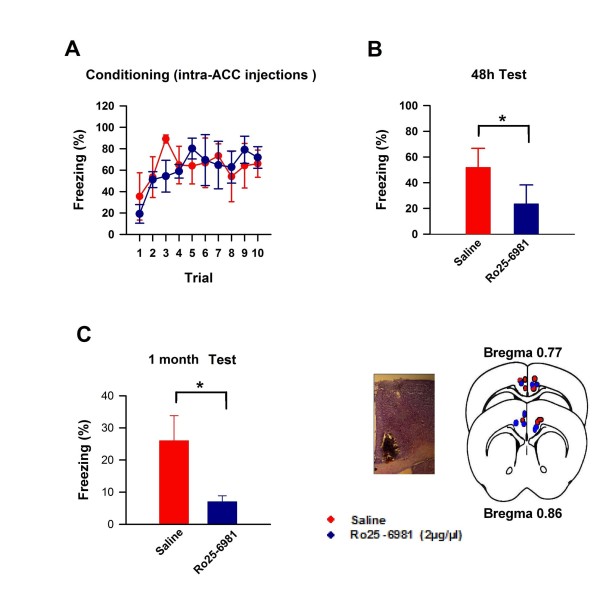
**GluN2B subunit activity within the ACC is necessary for trace fear learning**. **(A) **Mice exposed to bilateral 0.5 μl infusions into the ACC of either Ro25-6981 (2 μg/μl) or saline 15 minutes prior to trace fear training exhibited similar learning curves. **(B) **Mice exposed to Ro25-6981 prior to training showed a significant reduction in freezing behavior in response to the CS when tested in a new context 48 h later (saline: n = 4; Ro25-6981: n = 4, *t *= 3.06). **(C) (Left) **The memory impairment was still evident one month after training. **(Right) **Representative markers indicating microinjection locations. (* *P < 0.02*).

To determine if NMDA receptor GluN2B subunit activity within the ACC is necessary for trace fear learning, we implanted bi-lateral cannulae in the ACC of adult mice in order to administer pharmacological antagonists within this specific brain region [14]. After a two week recovery period, mice were exposed to bilateral 0.5 μl infusions of either Ro25-6981 (2 μg/μl) or saline 15 minutes prior to trace fear training. In accordance with our i.p. results, both groups showed similar learning curves (Figure 3A), but when tested in a new context 48 h later, mice exposed to Ro25-6981 prior to training showed a significant reduction in freezing behavior in response to the CS compared to mice exposed to saline (saline: 47 ± 4%; Ro25-6981: 18 ± 2%; *P *< 0.001; Figure 3B). In addition, to examine if this effect extended to remote fear memory, we also evaluated freezing behaviour one month after trace fear conditioning. Remarkably, the fear memory impairment was still evident when tested one month after training (saline: 26 ± 8%; Ro25-6981: 7 ± 2%; *P *= 0.002; Figure 3C). These results show that NMDA receptor GluN2B subunits within the ACC are necessary for trace fear learning, and suggest that long-term memory consolidation processes are disrupted by blocking early LTP-related mechanisms within the ACC during learning.

### CP-AMPAR activity within the ACC is necessary for trace fear memory consolidation

**Figure 4:**
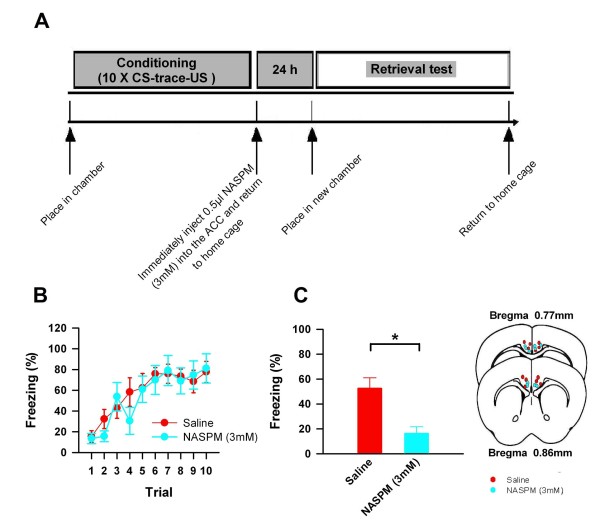
**ACC CP-AMPA receptors are necessary for trace fear memory**. **(A) **Immediately following trace fear conditioning, we exposed mice to bi-lateral, intra-ACC microinjections of either NASPM (3 mM, 0.5 μl/side) or saline (0.5 μl/side). **(B) **Learning curves. **(C) (Left) **When tested in a new environment 24 h later, mice exposed to intra-ACC injections of NASPM displayed significantly less freezing behavior in response to the CS (saline: n = 6; trace: n = 5, *t *= 2.51). **(Right) **Representative markers indicating microinjection locations. (* *P < 0.02*).

Our biochemical analysis revealed that trace fear conditioning rapidly upregulates postsynaptic AMPA receptor GluA1, but not GluA2, subunits within the ACC. Given that CP-AMPA receptors are GluA2 lacking, and are upregulated by *in vivo *experience in the mouse barrel cortex [26] and the lateral amygdala [27], we next sought to determine if CP-AMPAR activity within the ACC immediately following conditioning was necessary for long term memory consolidation. We exposed adult C57 mice implanted with bi-lateral cannulae over the ACC to the trace fear conditioning paradigm. Immediately after training we applied intra-ACC microinjections of the CP-AMPAR antagonist, 1-naphthylacetyl spermine (NASPM) (3 mM, 0.5 μl/side) or saline (0.5 μl/side; Figure 4A-B), and assessed memory retrieval twenty-four hours later in a new context. Remarkably, mice exposed to intra-ACC NASPM injections immediately after conditioning displayed significantly less freezing behavior in response to the CS than mice exposed to saline (saline: 53 ± 8%; trace: 16 ± 5%; *P *= 0.005; Figure 4C). In combination with our biochemical and behavioral data, these observations strongly indicate that trace fear learning is mediated through rapid, CP-AMPAR trafficking within the ACC that induce necessary subsequent memory consolidation processes.

### Trace fear learning rapidly induces functional CP-AMPA receptors within the ACC

**Figure 5:**
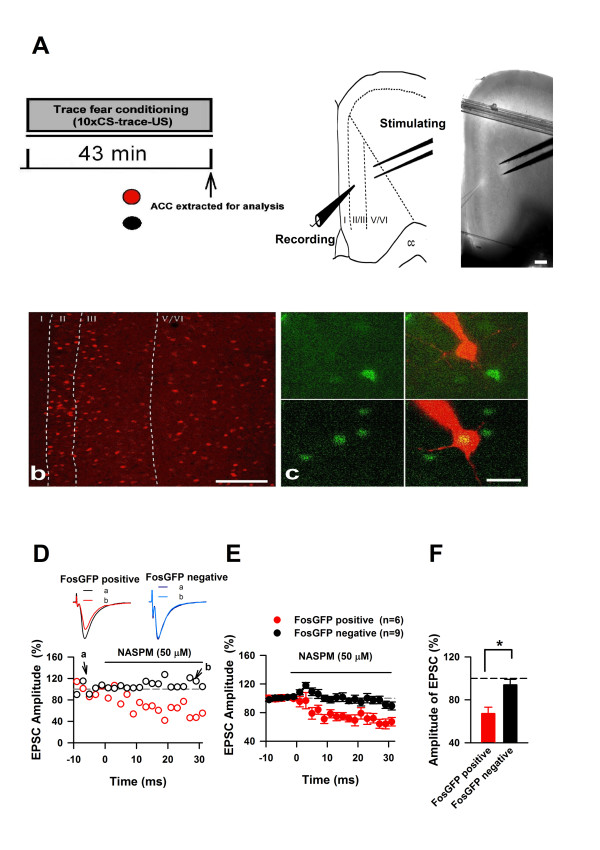
**Trace fear memory is mediated by postsynaptic CP-AMPAR trafficking within the ACC**. **(A) (Left) **We performed whole cell patch clamp recordings immediately following trace fear conditioning in ACC slices from transgenic FosGFP mice. **(Right) **We recorded EPSCs from pyramidal neurons in layer II/III whilst stimulating layers V/VI of the ACC. **(B) **Trace fear conditioning induces c-fos activity within the ACC; scale bar represents 100 μm. **(C) **Representative images of whole-cell patch clamp recordings of FosGFP negative pyramidal neurons (top panels), and FosGFP positive pyramidal neurons (bottom panels) as indicated by yellow showing overlap between GFP and dye loaded pipette; scale bar represents 20 μm. **(D) **Representative traces of single pyramidal neuron recordings before "a" and after "b" NASPM application. **(E-F) **NASPM significantly inhibited the amplitude of FosGFP positive neuronal EPSCs to 67% of the baseline, a reduction that is significantly greater than that observed on FosGFP negative neurons (FosGFP positive: n = 6; FosGFP negative: n = 9, *t *= -2.38).

In order to determine if the rapid membrane bound GluA1 upregulation observed within the ACC of mice exposed to trace fear conditioning corresponds to functional CP-AMPARs, we assessed the contribution of CP-AMPA channels to excitatory postsynaptic currents (EPSCs) observed in layer II/III pyramidal neurons through whole-cell patch-clamp recordings (Figure 5A). We used adult transgenic mice in which the expression of green fluorescent protein (GFP) is controlled by the promoter of the *c-fos *gene [14,26,28]. *C-fos *is an activity dependent gene that can be used as an indicator of recent neuronal activity [3,13], thus this method allowed us to record from recently activated neurons (FosGFP positive), whilst performing observations from neighboring neurons in the same slice that were not activated (FosGFP negative), thus allowing for robust within subjects comparisons (Figure 5B-C). Our previous work with these transgenic mice revealed that GFP expressing neurons within the ACC express changes in excitatory transmission in response to neuropathic pain, and that these changes are not present in GFP negative neurons [14]. We tested the effects of NASPM on EPSCs of FosGFP positive and FosGFP negative pyramidal neurons within the ACC of mice exposed to trace fear conditioning. Remarkably, we observed that NASPM significantly inhibited the amplitude of FosGFP positive neuronal EPSCs to 67% of the baseline (Figure 5D-E), an attenuation that was significantly greater than that observed on FosGFP negative neurons (FosGFP positive: 67.2 ± 5.9%; FosGFP negative: 93.95 ± 5.13%; *P = *0.005, Figure 5F). The increased NASPM sensitivity observed in ACC pyramidal neurons from trace fear conditioned mice indicates an increase in active CP-AMPA receptors, and shows that trace fear conditioning induces rapid CP-AMPAR trafficking within the ACC.

### Trace fear extinction eliminates NASPM induced attenuation of ACC pyramidal EPSCs

**Figure 6:**
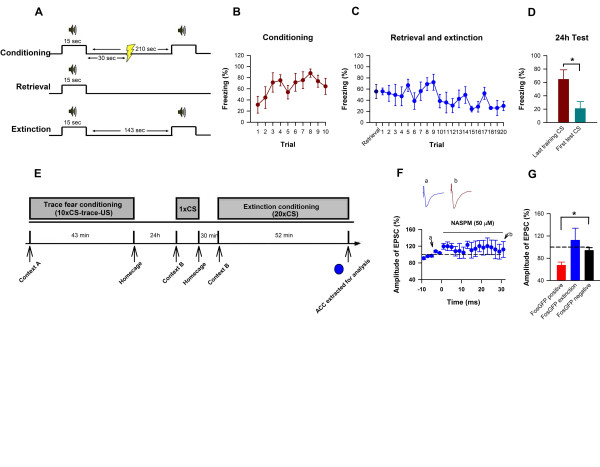
**Reconsolidation update eliminates NASPM sensitivity in ACC pyramidal neurons**. **(A) **Reconsolidation update protocol. **(B-D) **Reconsolidation update robustly extinguishes trace fear memory recall. **(E) **We extracted the ACC of mice immediately following reconsolidation update of trace fear memory. **(F-G) **Reconsolidation update abolishes NASPM sensitivity of layer II/III pyramidal neurons in the ACC induced by trace fear memory conditioning, (FosGFP positive extinction: n = 4; FosGFP negative: n = 9, *t *= -2.38). (G includes FosGFP positive conditioning mean from Fig 5 for comparison). (* *P < 0.02*).

The present findings indicate that trace fear conditioning is mediated through GluN2B dependent upregulation of synaptic, CP-AMPA receptors. Recently, a robust extinction protocol that can completely eliminate fear memory [29], named reconsolidation update (Figure 6A-D), has been found to induce the removal of CP-AMPA receptors in the lateral amygdala [27]. We thus next investigated the possibility that a similar mechanism within the ACC may mediate trace fear memory extinction. Twenty four hours following exposure to trace fear conditioning, we exposed adult FosGFP transgenic mice to reconsolidation update extinction conditioning and immediately removed the ACC for analysis (Figure 6E). Remarkably, this robust extinction paradigm completely eliminated the EPSC amplitude reducing effect of NASPM in FosGFP positive ACC pyramidal cells (FosGFP positive extinction: mean: 112.0 ± 21.9%; Figure 6F-G), showing that reconsolidation update extinction training can rapidly reduce the number of active CP-AMPA receptors within the ACC, suggesting that rapid synaptic CP-AMPA receptor induction and removal within the ACC mediates trace fear memory.

## Discussion

The present study is the first to demonstrate that rapid AMPA receptor potentiation within the ACC mediates trace fear learning. We have identified that trace fear conditioning induces an upregulation of membrane bound GluA1 within the ACC that is evident immediately after conditioning. We have shown that NMDA receptor GluN2B subunit activity within the ACC is critical for trace fear learning, and disruption of these receptors during conditioning prevents AMPA receptor GluA1 subunit upregulation and fear memory retrieval. Furthermore, we found that blockade of CP-AMPAR activity immediately following trace fear conditioning is sufficient to prevent trace fear memory retrieval 24 h later. Accordingly, through the use of transgenic FosGFP mice, we observed that trace fear learning potentiates CP-AMPARs in c-fos expressing ACC pyramidal cells. These findings show that early memory formation occurs within the cortex during trace fear learning, and identifies a critical, rapid synaptic strengthening mechanism that is necessary for consolidation of long term fear memory.

### Trace fear learning induces immediate membrane GluA1 upregulation

A key component of LTP induction is the upregulation of postsynaptic AMPA receptors [30], and AMPA receptor plasticity is strongly implicated in learning and memory [5,21]. Here we report a rapid increase in membrane GluA1 subunit protein in the ACC of mice extracted immediately after *in vivo *trace fear learning. This is consistent with recent observations that trace eye blink conditioning induces changes in neuronal firing within the mPFC [31]. Our findings support the notion that experience dependent synaptic activity can "tag" specific synapses for subsequent changes in excitatory transmission [32]. Previous investigations of fear learning have identified changes in excitatory transmission within the hippocampus and amygdala [4-7,33], and increases of GluA1 in dendritic spines of CA1 neurons have been observed 24 h after contextual fear conditioning [33]. Our results therefore suggest that fear learning is mediated through a complex interplay between various brain areas, and that rapid plasticity within the cortex is in itself a mediator of learning induced alterations that are required for long term memory consolidation. Indeed, the recent findings that CaMKIV is required for translation-dependent early synaptic potentiation within the ACC [34] and that trace fear memory is enhanced in mice overexpressing CaMKIV [12], suggest that targeting these early cortical changes induced by learning can alter the strength of the consolidation of the fear memory.

### NMDA receptor GluN2B subunit dependent AMPA GluA1 upregulation

We found that *in vivo *blockade of NMDA receptor GluN2B subunits during trace fear conditioning prevented fear memory recall, and blocked the upregulation of membrane bound GluA1; indicating that GluA1 subunits are the downstream target of experience dependent GluN2B activity. This is in accordance with previous observations that genetic GluN2B overexpression can enhance fear memory acquisition [20]. Although LTP has long been considered to be the neural substrate for learning and memory [2], and reports have shown that NMDA GluN2B subunit activity is critical for ACC LTP [35], and that AMPAR insertion corresponds to potentiation of excitatory synaptic transmission [22,36,37], this is the first evidence that *in vivo *trace fear learning induces rapid GluN2B mediated AMPAR insertion within the cortex. Indeed, although various publications implicate NMDA receptors in several brain regions in fear memory, including the amygdala [25], hippocampus [38,39], and forebrain [19], studies had yet to identify the learning related downstream target. In addition, studies have questioned the requirement of NMDA GluN2B receptors in hippocampal LTP and learning [40,41]. Our findings highlight that there is a critical cortical contribution to fear learning, and that early GluN2B dependent plasticity within the cortex is necessary for long term memory recall.

### CP-AMPAR activity within the ACC is necessary for memory consolidation

Mounting evidence indicates that AMPA receptor trafficking is a critical component of synaptic strengthening, and may underlie learning [21,30,42]. Accordingly, we observed that *in vivo *blockade of CP-AMPARs in the ACC immediately following trace fear conditioning robustly blocked memory retrieval 24 h later. In combination with our biochemical and behavioral data, these findings indicate that rapid CP-AMPA receptor upregulation during conditioning is necessary for long term memory consolidation, and is the first account that early memory formation within the ACC is necessary for long term retrieval. Several studies support the recruitment of CP-AMPARs in synaptic strengthening [22-24]. In particular, in the CA1 region of the hippocampus, different LTP induction protocols have been demonstrated to induce transient increases of CP-AMPARs, including theta burst stimulation [23], and pairing protocol [24], but see [43]. Of particular interest are previous *in vitro *observations in the CA1 region of the hippocampus, where LTP induced by a pairing protocol corresponded to rapid CP-AMPAR upregulation [24] that lasted less than 25 minutes, and was NMDA receptor dependent. Importantly, LTP induction corresponded to increases in sensitivity to the CP-AMPAR channel blocker polyamine toxin philanthotoxin 433 (PhTx). Remarkably, there was a lack of LTP recovery if PhTx was applied immediately after LTP induction, suggesting that activation of new CP-AMPARs immediately after LTP induction is necessary for subsequent LTP. Here we present *in vivo *evidence that disrupting CP-AMPAR activity within the ACC immediately after conditioning prevents subsequent memory consolidation processes necessary for long term memory retrieval. Thus our findings support the notion that activity-dependent synaptic "tagging" may mediate stabilization of LTP [32], and that such mechanisms within the cortex are rapidly engaged during trace fear learning.

### Recording from fear-triggered ACC neurons

Several studies indicate that memory storage for a given memory is mediated by a select population of neurons [5,22,44]. Given that various publications have suggested correlations between c-fos expression and synaptic strengthening and learning [13,22,33], we used FosGFP transgenic mice to assess the effects of trace fear conditioning on CP-AMPAR mediated currents. Using whole cell patch clamp recordings of recently activated pyramidal neurons in layer II/III of the ACC, we observed that blockade of CP-AMPARs yielded a reduction of EPSC amplitude to 67% of baseline, significantly greater than in neighbouring FosGFP negative cells, confirming that trace fear conditioning potentiates postsynaptic CP-AMPAR activity. Interestingly, we observed that reconsolidation update extinction training completely reverses this effect, indicating that fear memory is mediated through CP-AMPAR trafficking within the ACC.

We focused our recordings on layer II/III neurons as there is strong evidence that thalamic-ACC evoked potentials extend through layer V/VI of the ACC and into layer II/III [45]. Furthermore, we have previously shown that within the ACC, pyramidal neurons in layer II/III undergo changes in excitatory transmission in response to LTP induction protocols and chronic pain [14,15,20]. More importantly, through recordings of layer II/III pyramidal ACC neurons, we identified enhanced LTP in mice overexpressing CaMKIV, which corresponded with enhancements in trace fear learning [12]. Furthermore, although observations within the amygdala have shown that fear memory extinction is mediated through CP-AMPAR removal [27], and postsynaptic GluA2 expression is required for fear memory maintenance [7], this is the first report that fear learning corresponds with *cortical *synaptic CP-AMPAR trafficking. Importantly, although CP-AMPARs are present in interneurons, it is unlikely that our recordings are affected by inhibitory transmission, as picrotoxin was present in all our recordings.

## Conclusions

**Figure 7:**
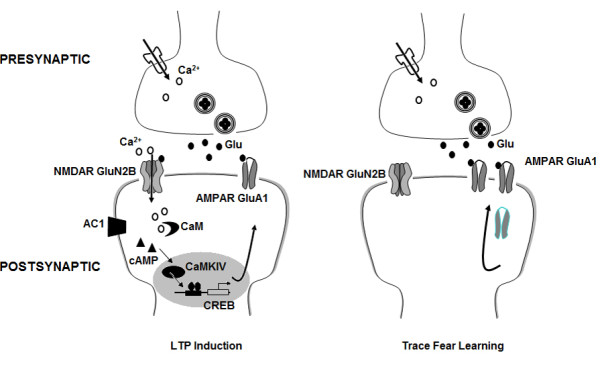
**Rapid AMPAR upregulation contributes to trace fear learning**. During trace fear conditioning, Ca^2+ ^influx via NMDARs initiates AMPAR upregulation through activation of Ca^2+^/calmodulin-dependent protein kinase IV (CaMKIV) related pathways. Newly recruited postsynaptic AMPARs help potentiate glutamatergic excitatory transmission within the ACC, establishing plasticity in neuronal populations for long term memory storage.

In summary, our findings indicate that early reorganisation within the ACC is critical for trace fear memory consolidation. Our findings suggest that trace fear learning is mediated through rapid excitatory potentiation within the ACC, and supports the notion that experience dependent synaptic activity can "tag" specific synapses for subsequent changes in excitatory transmission [32]. Furthermore, our results present strong evidence that such rapid potentiation is necessary for consolidation, suggesting that experience induced CP-AMPAR activity mediates memory stabilization within the cortex. Consolidation theory suggests that learning induces an initial rapid and transient strengthening of the connections between the hippocampus and cortical areas, whilst alterations of cortico-cortical connections are slower but long-lasting [46]. As many neurons in layer II/III within the ACC receive projections from layer V/VI neurons [45], our results suggest that rapid strengthening of cortico-cortical connections can occur. Furthermore, it is unlikely that diffuse hippocampal projections could be responsible for the detectable changes observed through stimulation of layer V-VI. We propose that during trace fear conditioning, the ACC is actively engaged and undergoing NMDA receptor driven AMPA receptor reorganisation (Figure 7). This reorganisation in turn provides a "tag" that will further facilitate plasticity related mechanisms necessary for the appropriate long term consolidation processes to occur, thus completely forming the trace fear memory.

## Methods

### Animals

Experiments were performed with adult (8-12 week) male C57/BL6 mice purchased from Charles River (Quebec, Canada) or transgenic FosGFP mice obtained from the laboratory of Dr. Alison Barth (Carnegie Mellon University). Animals were housed under a 12 h light/dark cycle, and all experiments were performed under protocols approved by the University of Toronto Animal Care Committee.

### Fear conditioning

All conditioning was completed in an isolated shock chamber (Medical Associates, St Albans, VT, USA). Trace fear conditioning was performed as reported previously [12]. Briefly, the conditioned stimulus (CS) used was an 80-db white noise, delivered for 15 s, and the unconditioned stimulus (US) was a 0.75-mA electric foot-shock for 0.5 s. Mice were acclimated for 5 min, and were presented with 10 trials in the following order: CS - trace - US - intertrial interval (ITI) (trace period = 30 s, ITI = 210 s). For delay fear conditioning, the conditioning stimulus (CS) used was an 80-db white noise, delivered for 15 s, and the unconditioned stimulus (US) was a 0.75-mA electric foot-shock for 0.5 s that was presented at 14.5 sec into the CS presentation, such that the CS and US co-terminated. Reconsolidation update conditioning was performed as published previously with minor modifications [29]. Briefly, 24 h following exposure to trace fear conditioning, mice were placed in a novel environment and exposed to one presentation of the CS; were then returned to their home cage for 30 min; and were then reintroduced to the novel environment and exposed to 20 CS presentations with an ITI of 143 sec. For memory retrieval tests, mice were introduced to a novel chamber and were acclimated for 5 min and subjected to a presentation of the CS to test for trace fear memory (Huerta, 2000 #210). All data were recorded using the video-based Freeze Frame fear conditioning system and analyzed by Actimetrics Software (Coulbourn Instruments, Wilmette). Average freezing for the baseline and for the trace period (30 s) following the CS during the training and testing sessions were analyzed. Freezing bouts of 1 s or more were considered as freezing (the absence of movement aside from respiration).

### ACC cannulae implantation and microinjection

We implanted bi-lateral cannulas into the ACC of mice as reported previously [18]. Briefly, mice were anaesthetized by intraperitoneal (IP) injections of a mixture of 1.3 mL of ketamine (100 mg/ml, Bimeda MTC, Cambridge, Ontario) and 0.5 ml of xylazine (20 mg/ml, Bayer, Toronto, Ontario, Canada) in 8.2 ml of normal saline at a dose of 10 μl per gram body weight. Mice heads were secured on a stereotaxic frame and 24-gauge guide cannulas were implanted bilaterally into the ACC (0.7 mm anterior to bregma, ± 0.3 mm lateral from the midline, 0.9 mm beneath the surface of the skull). Mice were given 2 weeks to recover after cannula implantation. Intra-ACC injections were delivered via a 30-gauge injection cannula that was lowered 0.85 mm further into the brain than the guide. The microinjection apparatus consisted of a Hamilton syringe (10 μl) connected to an injector needle (30 gauge) by a thin polyethylene tube and motorized syringe pump. All infusions consisted of 0.5 μl of solution delivered at a rate of 0.05 μl/min. Injection sites were confirmed at the end of all experiments and sites outside of the ACC region were excluded from the study.

### Membrane preparation

Membrane preparation was performed as previously described [47] with minor changes. Briefly, ACC samples were dissected in cold D-PBS and resuspended in Buffer 1 (2 mM Tris-EDTA, 320 mM sucrose, 5 mM MgCl_2_, and 1X protease inhibitor cocktail, pH 7.4), and homogenized. Each sample was centrifuged at 1000 × g for 10 min and the supernatants (S1) were recovered. The remaining pellet (P1) was then resuspended in Buffer 2 (50 mM Tris-HCl, 2 mM Tris-EDTA, 5 mM MgCl_2_, and 1X phosphatase inhibitor cocktail 1 and 2, pH 7.0) and centrifuged at 1000 × g for 10 min, with its supernatant (S2) collected and combined with S1. The remaining pellet (P2) was resuspended in Buffer 2, and again centrifuged at 1000 × g for 10 min., and its supernatant (S3) was combined with S1 and S2. Combined supernatant fractions (S1, S2 and S3) were finally centrifuged at 39,000 × g for 30 min, the resulting supernatant contained the cytosolic fractions, and the resulting pellet (membrane fractions) was resuspended in Buffer 3 (50 mM Tris-HCl, 2 mM Tris-EDTA, 3 mM MgCl2, and 1X phosphatase inhibitor cocktail 1 and 2, pH 7.4).

### Western blot analysis

Western blot was performed as previously described [48]. Sample protein concentrations were quantified using Bradford assay, and electrophoresis of equal amounts of protein was performed on NuPAGE 4-12% Bis-Tris Gels (Invitrogen, Carlsbad, CA). Separated proteins were transferred to polyvinylidene fluoride membranes (Pall Corporation, East Hills, NY) at 4°C overnight for analysis, and were then probed with primary antibodies as follows: anti-GluA1 (1:4000, rabbit polyclonal), anti-GAPDH (1:6000, mouse monoclonal), anti-GluA2/3 (1:1000, rabbit polyclonal), followed by horseradish peroxidase (HRP)-coupled secondary antibody diluted at 1:3000 for 2 hours followed by enhanced chemiluminescence detection of the proteins with Western lightning chemiluminescence reagent plus (PerkinElmer Life Sciences). ImageJ software (National Institute of Health) was used to assess the density of immunoblots by a blind observer.

### Immunohistochemistry

Immunostaining was performed as described previously [12,14]. Briefly, mice were anesthetised with isoflurane and perfused with 0.01 mol/l phosphate-buffered saline (PBS; pH 7.4) via the ascending aorta followed by perfusion of 4% paraformaldehyde (PFA) in 0.1 mol/l PB at 4°C. The brains were removed and post-fixed for 4 hours in 4% PFA, after which brains were placed in vials filled with 30% sucrose in 0.1 mol/l PB overnight at 4°C for at least 48 hours, or until the brain fully dropped to the bottom of the jar. Brain sections containing the ACC were cut using a cryostat (Leica) at 30 μm thickness. Briefly, sections were sequentially incubated through the following solutions: (i) a solution of 3% bovine serum albumin (BSA; Sigma, St Louis, USA) and 0.3% Triton X-100 containing anti- c-fos (1:500 abcam) primary antibody for 3 days at 4°C. (ii) Biotin labelled goat anti-rabbit secondary antibody (1:1000 Santa Cruz, CA) for 24 hours at 4°C (iii) Cy3 conjugated streptavidin (1:1000; Santa Cruz, CA, USA) for 2 hours at room temperature. In between each step, sections were rinsed with PBS 3 times for 10 min. Sections were mounted on gelatin coated slides, air-dried, cleared and cover-slipped for observation under a confocal microscope (FV-1000, Olympus, Japan).

### Electrophysiology

Coronal brain slices (300 μm) at the level of the ACC were prepared using standard methods [14,15,49] immediately after trace fear conditioning. Slices were transferred to a submerged recovery chamber with oxygenated (95% O_2 _and 5% CO_2_) artificial cerebrospinal fluid (ACSF) containing (in mM) 124 NaCl, 2.5 KCl, 2 CaCl_2_, 1 MgSO_4_, 25 NaHCO_3_, 1 NaH_2_PO_4_, and 10 glucose at room temperature for at least 1 hr. Experiments were performed in a recording chamber on the stage of a BX51W1 microscope equipped with infrared differential interference contrast optics for visualization. Excitatory post-synaptic currents (EPSCs) were recorded from layer II/III neurons with an Axon 200B amplifier (Molecular Devices Inc., Sunnyvale, CA, USA), and the stimulations were delivered by a bipolar tungsten stimulating electrode placed in layer V of the ACC. AMPA/KA receptor-mediated EPSCs were induced by repetitive stimulations at 0.05 Hz, and neurons were voltage-clamped at -60 mV (without liquid junction potential correction) in the presence of AP5 (50 μM). The recording pipettes (3-5 MΩ) were filled with a solution containing (in mM) 124 K-gluconate, 5 NaCl, 1 MgCl_2_, 0.2 EGTA, 10 HEPES, 2 Mg-ATP, 0.1 Na_3_-GTP, and 10 phosphocreatine disodium (adjusted to pH 7.2 with KOH). Picrotoxin (100 μM) was always present to block γ-aminobutyric acid (A) (GABA_A_) receptor-mediated inhibitory synaptic currents in all experiments. The initial access resistance was 15-30 MΩ, and it was monitored throughout the experiment. Data were discarded if the access resistance changed > 15% during an experiment. Data were filtered at 1 kHz, and digitized at 10 kHz.

### Drugs

In order to block NMDA receptor GluN2B subunit activity, we used Ro-25-6981 or Ifenprodil (Tocris Bioscience), NMDA receptor antagonists that target the GluN2B subtype [18,50]. Mice were given i.p. injections of 10 mg/kg doses, and bilateral ACC 0.5 μl infusions (2 μg/μl) for the microinjection studies. To block CP-AMPA receptors we used the antagonist, 1-naphthylacetyl spermine (NASPM).

### Nociceptive behavioral tests

In the hotplate test, mice were placed on a standard thermal hotplate with a heated surface (55°C) (Columbus Instruments, Columbus, OH). The latency for nociceptive responses was recorded with a cut-off time of 30 seconds. The spinal nociceptive tail-flick reflex was evoked by radiant heat (Columbus Instruments, Columbus, OH) applied to the underside of the tail, and latencies were measured with a cut-off time of 10 seconds.

### Data Analysis

Results are expressed as mean ± SEM. Statistical comparisons were made using a one way ANOVA adjusted by the Holm-Sidak test for multiple comparisons, or unpaired student *t*-tests. In all cases, *P *< 0.02 is considered statistically significant.

## Competing interests

The authors declare that they have no competing interests.

## Authors' contributions

GD, VM, and MZ conceived of the project and designed experiments. GD and VM performed biochemical experiments. GD performed behavioral experiments, analyzed data and wrote the manuscript. XYL, CT, and KK designed and performed electrophysiological recordings. All authors read and approved the manuscript.
